# Correlation between dental caries experience and mutans streptococci counts using saliva and plaque as microbial risk indicators in 3-8 year old children. A cross Sectional study

**DOI:** 10.4317/jced.51814

**Published:** 2015-02-01

**Authors:** Jasmine Nanda, Vinod Sachdev, Meera Sandhu, Kanwar Deep-Singh-Nanda

**Affiliations:** 1 Post Graduate Student, Department of Pedodontics and Preventive Dentistry, I.T.S Centre for Dental Studies and Research, Ghaziabad; 2 MDS, Department of Pedodontics and Preventive Dentistry, I.T.S Centre for Dental Studies and Research, Ghaziabad

## Abstract

Objectives: Determination of the relative amounts of mutans streptococcus in both saliva and plaque and to study its correlation with dental caries in children. Study Design: The study comprised of 60 children aged 3-8 years divided into 2 groups (30 children in each): Group A- Children with more than 4 carious teeth and Group B- Children without caries. Saliva and plaque was collected from children of both the groups with the help of Dentocult SM strip test kit (Orion Diagnostic). Following incubation, mutans streptococcus scores (from 0 to 3) in each individual was evaluated and compared between both the groups. Results: On comparing the two groups, mean ± SD of saliva score and plaque score was 2.40 ± 0.675 and 2.40 ± 0.621 respectively in group A, whereas it was 0.60 ± 0.498 and 0.83 ± 0.531 in children of group B showing a significant correlation (p = < 0.001) between mutans streptococci scores in both saliva and plaque and dental caries experience. Conclusions: There is a direct and strong co-relation between the salivary and plaque mutans streptococcus counts and caries activity in children aged 3-8 years.

** Key words:**Mutans streptococci, dentocult, dental caries.

## Introduction

Dental caries as well as periodontal diseases have been considered as the most important global oral health burdens. According to the WHO bulletin, 60-90% of school aged children of most of the industrialized countries are affected by dental caries. This major health problem reduces the quality of life of an individual, causing pain and suffering as well as impairment of function. Though the levels of dental caries in most of the developing countries, like India were low until recent years, the prevalence rates of dental caries experience are now tending to increase (1). Despite the advances in preventive dentistry, dental caries stands out as one of the most prevalent biofilm – mediated diseases affecting humans.

The mechanisms of dental caries is well established to the point where new approaches are being made for caries prevention based on scientific understanding of the processes involved. Dental caries can be prevented or arrested at an early stage using several methodologies available for caries risk assessment. The number of mutans streptococci in saliva can be used for the evaluation of caries risk and also for monitoring the level of microbial colonization in children.

Streptococcal species have been established as the major cariogenic bacterial species. Mutans streptococci and S. sobrinus are the common streptococcal (MS) species recovered from human oral microflora. In individual subjects, the occurrence of S. mutans is normally higher than that of S. sobrinus. Their acquisition and high counts leads to high frequency of dental caries in children (2). Since, the maximum colonization of mutans streptococcus already gets established by mixed dentition stage, therefore microbial screening of children is most appropriate at this early age group.

A variety of methods of microbial tests for caries risk assessment have been used for the identification of oral mutans streptococcus, other than conventional culture methods, such as quick simple chair side tests, computerized caries risk assessment (cariogram), direct enzyme tests, Enzyme-linked immunosorbant assays, PCR, etc. The use of these technologies will require extensive retraining of clinical dentists. Since, these advanced techniques require extensive training and high cost, therefore a chair-side caries activity test such as mutans streptococci test can be used as a motivating factor as well as a useful tool in diagnosing and formulating successful treatment plan for effective preventive programme in children.

Dentocult SM is a commercially available kit used in clinical or epidemiological studies and is able to detect and evaluate MS, conveniently, at the chair side, without expensive equipments. One of the most important advantages of this test is that the test strips can be preserved for future and can be used as a motivational tool for both the patient and their parents.

Therefore, the present study is aimed at evaluating the levels of mutans streptococci in saliva and plaque in 3-8 year old children with and without caries as well as evaluating the feasibility of using Dentocult SM strip test as a chair side caries activity test as well as a motivational tool.

## Material and Methods

The present study was conducted in the Department of Pedodontics and Preventive Dentistry at I.T.S Centre for Dental Studies and Research, Muradnagar, Ghaziabad.

Inclusion Criteria:

1. Children having more than 4 carious lesions (Group A).

2. Children having no caries ( Group B).

Exclusion criteria :

1. Physically or mentally handicapped children.

2. History of antibiotic therapy or fluoride treatment in the past 2-4 weeks.

3. Children undergoing any kind of interceptive orthodontic treatment.

Sample selection.

60 children aged 3-8 years who came to the Department of Pedeodontics and Preventive Dentistry, at ITS Centre for Dental Studies and Reasearch, were selected for the study.

Group division:

60 children who came to the Department of Pedeodontics and Preventive Dentistry, at ITS Centre for Dental Studies and Reasearch, were randomly selected for the study. They were divided into two groups A and B, each comprising of 30 children.

Group A: 30 children (11 females and 19 males) with more than 4 carious teeth

Group B: 30 children ( 14 females and 16 males) without caries.

-Methodology:

Ethical committee approval was taken prior to the study by the ethical committee of the institution. Informed consent was taken from all the parents before the start of the study.

-Armamentarium.

Mouth mirror; Probe; Explorer; Tweezer; Dentocult SM strip test kit(Orion Diagnostic, Finland) comprising of:- A) 10 Strips for plaque.; B) 10 Strips for saliva; C) Selective culture vials; D) Bacitracin discs; E) Paraffin pellets; F) Patients label; G) Plastic pouch.

-Clinical examination:

It was a blind study. The clinical examination was performed on the dental chair with optimal light. All the subjects were instructed to refrain from tooth brushing and eating for 2 hours before the sample collection. No radiographs were taken. Children with visible carious involvement were selected in group A.

-Oral microbiological sampling and interpretation:

The saliva and plaque samples were collected from children of both the groups using Dentocult SM strip test kit. Fifteen minutes before taking the sample, a bacitracin disc was placed in the selective culture broth. The bacterial sampling was carried out as follows:

A) Saliva sampling.

Since most of the children were too young to safely chew paraffin, therefore unstimulated saliva sample was obtained by pressing the rough surface of the strip against the patient’s tongue. The strip was then removed through the patient’s gently closed lips.

B) Plaque sampling:

Plaque was collected using toothpick from an interproximal site. Collected plaque was spread thoroughly but gently over the rough surface of the square-tipped strip. Four sites were simultaneously sampled.

The selective culture vial was gently shaken for the even distribution of bacitracin. The strips were placed with the smooth surfaces clipped and attached to the cap, in the selective culture broth. The vials were incubated in an upright position at 35 ˚ - 37 ˚ C for 48 hours with the cap one quarter of a turn open.

-Interpretation:

Interpretation was done by another observer who was unaware of the caries status of the subjects. The presence of mutans streptococcus was evident by the dark blue to light blue, raised colonies on the inoculated surface of the strip. Mutans streptococci colonies were differentiated from the colored plaque debris in being clearly elevated from the strip surface. According to the Dentocult Kit manufacturer’ model chart, scores were given as 0, 1, 2 and 3 based on the density of the growth ( Table 1).

**Table 1:**
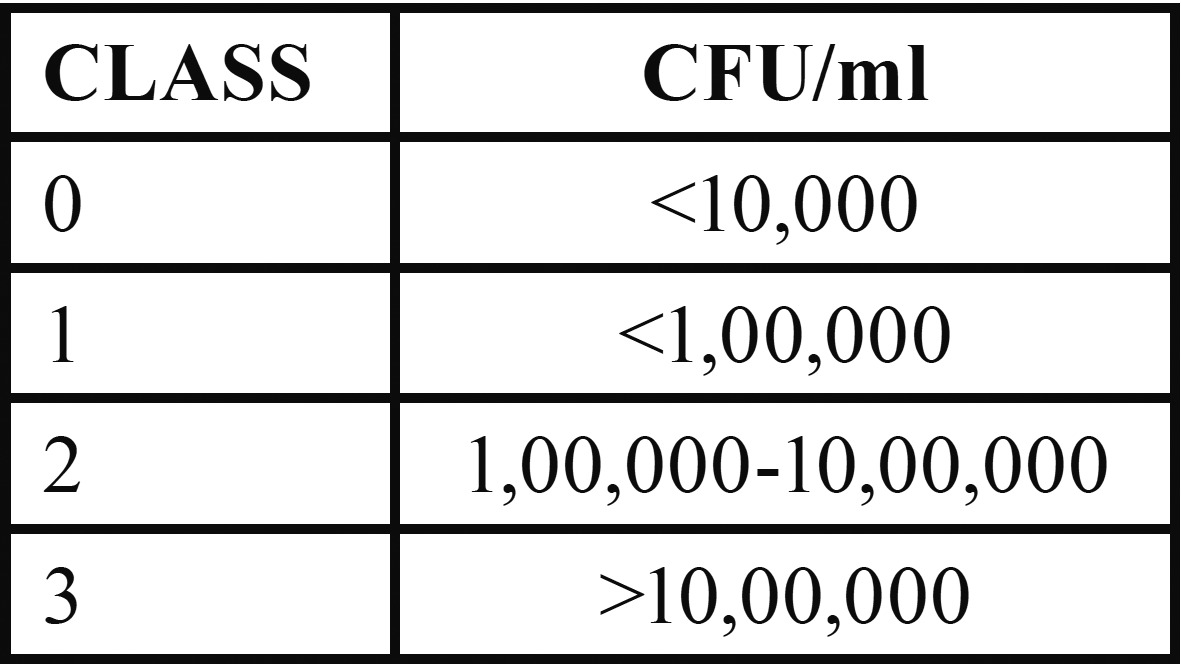
Model chart.

-Statistical procedures used.

Data were analyzed using the SPSS 16 software. Spearman’s coefficient test was used to test the correlation between the dental caries experience and bacterial counts in plaque and saliva scores in each of the groups (group A and group B).

Mann Whitney test was used for the comparison of saliva scores and plaque scores between both the groups.

## Results

In this study, Group A consisted of 30 children, out of which 11 were females and 19 were males with more than 4 carious teeth. Group B consisted of 30 children, 14 females and 16 males without dental caries. Mutans streptococci counts in saliva and plaque samples from each groups was evaluated with the help of Dentocult SM strip mutans test kit. The results of the study are as follows: ( Table 2).

**Table 2:**
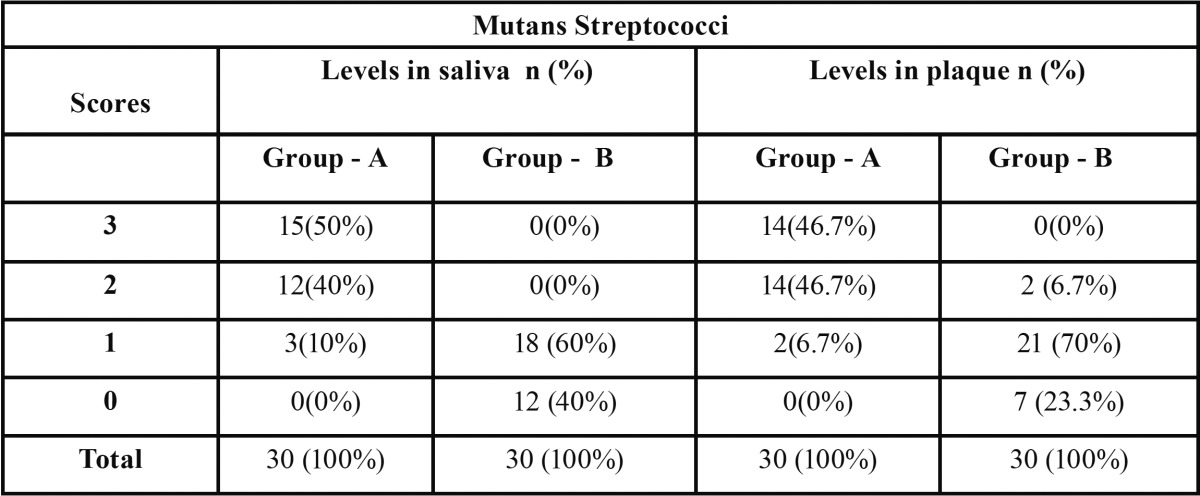
Comparison of Mutans streptococci scores in saliva and plaque of children with caries (group A) and children without caries (group B).

-Group A.

Majority of the children i.e. 50% and 46.7% (in their saliva and plaque respectively) demonstrated mutans streptococci score of 3 (more than 106 CFU/ml); showing a positive correlation between mutans streptococci scores in both saliva and dental plaque (Spearman’s Correlation Coefficient = 0.481; *p*= 0.007 ).

-Group B.

Majority of the children i.e. 60 % and 70% (in their saliva and plaque respectively) showed score 1 (less than 10 5 CFUs/ml). However, the corelation between saliva and plaque score in group was not statistically significant (*p* > 0.05) in group B ( Table 3).

**Table 3:**
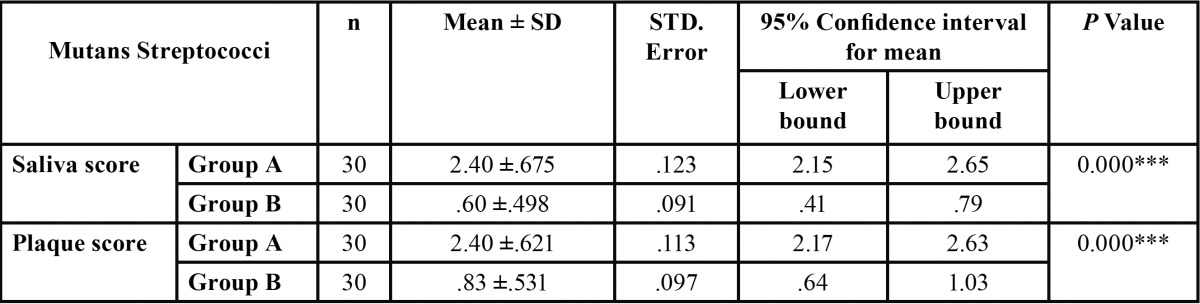
Mean scores of mutans streptococci in Saliva and plaque in children with and without caries (group A and group B).

On comparing both the groups, mean ± SD of saliva score was 2.40 ± 0.675 in children of group A, whereas it was 0.60 ± 0.498 in group B children (*p* = < 0.001). Also, the mean ± SD of plaque score was 2.40 ± 0.621 in children of group A, whereas it was 0.83 ± 0.531 in children without caries (*p* = < 0.001). Thus, it demonstrates a strong correlation between dental caries experience and the mutans streptococci scores found in both saliva as well in plaque in 3-8 year old children ( Table 4).

**Table 4:**
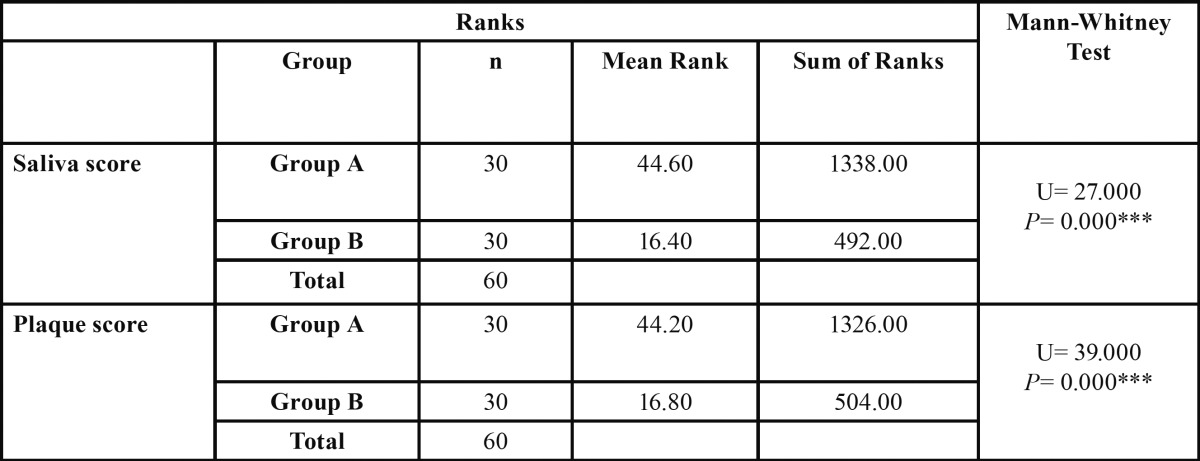
Comparison of the mean ranks of saliva and plaque scores in children with and without caries (Group A and Group B).

Mean ranks of saliva score of children in group A was 44.60, which was significantly higher than that found in children in group B which was 16.40. The mean rank of plaque score of children in group A was 44.20, which was significantly higher than that found in children in group B which was 16.80. On applying Mann- Whitney test for inter group comparison, the value for saliva score was found to be 27.000 and for plaque score it was 39.000. There was a highly significant relation between both the groups as *p* < 0.001. Thus, it shows that samples from both saliva as well as plaque are important microbial risk indicators of mutans streptococcus in children aged 3-8 years.

## Discussion

Dental caries is a preventable disease, therefore, preventive strategies are required for children who are considered to be at risk for caries development such as those with active lesions as well as those with proven caries susceptibility. Assessment of caries risk status also provides an estimate of future caries activity. Hence, an accurate caries risk assessment is a necessary prerequisite to formulate a strategy for preventing dental caries.

In the present study children aged 3-8 years have been selected because stimulated saliva is difficult to obtain in the children below 3 years. Primary dentition is fully established at 3 years in most of the children. So, at this age all the primary teeth surfaces are assumed to be colonized by mutans streptococcus. Permanent teeth start erupting in the oral cavity at about 6 years of age and bacterial colonization of new surfaces of these teeth begins. Thus, caries risk assessment, prevention and motivation of children should be initiated in this age group.

In this study, there was a significant corelation between the mutans streptococci counts present in saliva of caries active children and dental caries (*p*= 0.007). These findings are similar to other studies by Lindquist and Emilson (3) that indicated that there is a positive relationship between prevalence of mutans streptococci in saliva and its colonization on the dorsum of the tongue (*p*< 0.001). Fujiwara *et al.* (4) also found a significant correlation between the number of mutans streptococci in saliva and the caries prevalence and also with caries initiation in future (*p*= 0.0001).

In the present study, interproximal plaque was used for sampling. The interproximal sites of the molars provide an area for stagnation for refined carbohydrates. Thus, mutans streptococci is most frequently found in the proximal areas. This has been supported by the study done by Seki *et al.* in 2003 (5) in which the mutans streptococci estimation in plaque showed a sensitivity of 70% proving that dental plaque is an appropriate site for estimating Mutans species .In the present study also, there was a direct relationship of mutans streptococci levels found in dental plaque and dental caries in children. Hence, we can say that density of mutans streptococci in dental plaque is also a good indicator of caries risk.

However, in group B it was observed that not all the non carious subjects had low mutans streptococci counts (*p*= 0.536). This can be explained by the fact that since dental caries is multifactorial, therefore not all mutans streptococci infected children are expected to have caries. It can be considered that though the children in group B did not show dental caries, but they possess atleast one essential component of the caries process (bacterial component). Thus, they are also considered at risk than the uninfected children in the same group. Also, the possibility of missing the incipient lesions might have contributed to the weak association.

On comparing the bacterial counts in both the groups, it was found that the mutans streptococci colony forming units in caries active subjects was higher in both saliva and plaque samples as compared to that found in caries free subjects. The mean saliva score of mutans streptococci in caries active children was 2.40 ± 0.675. Whereas, in caries free children it was observed to be 0.60 ± 0.498, resulting in a highly significant difference as *p* = 0.000. With respect to plaque score of mutans streptococci in caries active children, the mean score was found to be 2.40 ± 0.621. Whereas, in caries free children the mean score was 0.83 ± 0.531 showing a highly significant difference between both the groups (*p*= 0.000).

Based on the above findings, it can be observed that there is a strong corelation between mutans streptococci counts and dental caries. Similar findings were observed by Neeru Singh *et al.* in 2010 (6) using the Dentocult kit. This has also been supported by Corby *et al.* in 2005(7) which showed the overabundance of mutans streptococci species in 90% of caries active subjects. Whereas, in a study conducted by Becker *et al.* in 2002 (8), the results indicated that Actinomyces species were found to be highest in number in childhood caries and have a major role in caries initiation.

In this study, using Dentocult test, a positive and a highly significant corelation was found between the saliva and plaque streptococcus counts and caries activity (*p* value = 0.007) in caries active children. After the development of Dentocult SM strip test by Jensen and Bratthall in 1989 (9), there have been many studies investigating its use in pediatric patients. Seki *et al.* in 2003 (5) evaluated the clinical ability of Dentocult SM strip test to identify preschool children at caries risk by evaluating mutans streptococci counts in their saliva and plaque. It was found to have a high prediction value of 80% and 71% in mutans counts in plaque and saliva respectively. Sizhen Shi *et al.* in 2003 (10) compared the efficiency of three caries activity tests ( Dentocult SM, Dentocult LB, Dentobuff strip) in predicting caries risk. Dentocult SM strip test was found to be the best amongst the others.

It is a useful and a quick method for the evaluation of caries risk. It is a chair side method, takes less time and can simultaneously detect mutans streptococci from plaque and saliva samples both. Moreover; it does not require complex expensive equipments. In a study by Twetman in 1994 (11), the results indicated that salivary mutans streptococci test is a motivational factor and a preventive strategy in children. However, there are certain limitations of this kit. Certain contaminating bacteria like enterococci and staphylococci may turn the selective culture broth brownish, violet or turbid, resulting in colorless mutans streptococci growth. In such cases, a new sample has to be taken (as suggested by the manufacturer).

Treatment for caries is a stressful and painful experience for the child as it often requires multiple visits, extensive restorations and extractions. Moreover, it becomes quite expensive for the parents and adds problems to those living in the developing countries. Predictive and precise caries risk assessment, that is less time consuming, allows the clinician to identify problems at an early stage. For a pediatric dentist, Dentocult chair side test can help in deciding rationally not only on the preventive therapies, but also on the treatment objectives according to a children’s level of risk; including frequency of recalls, counseling and motivating the parents and the child on the preventive methods.

Thus, the present study concludes that i) Mutans streptococci counts were higher in saliva and plaque in the children with caries as compared to caries free children demonstrating a significant corelation between the plaque and salivary mutans streptococci counts and dental caries. ii) Density of mutans streptococci in both saliva and dental plaque are a good indicator of caries risk. iii) Dentocult SM strip test is a quick and a reliable indicator test for caries activity as well as a useful motivational tool for children as well as for parents.
